# Coevolution of specific gut microbiota of Min pig with host cold adaptation through enhanced vitamin B1 synthesis

**DOI:** 10.3389/fmicb.2024.1448090

**Published:** 2024-08-30

**Authors:** Yang Chang, Ziwen Zhang, Jiancheng Cai, Chunan Wang, Di Liu, Zhonghua Liu, Chunzhu Xu

**Affiliations:** ^1^Key Laboratory of Animal Cellular and Genetic Engineering of Heilongjiang Province, Northeast Agricultural University, Harbin, China; ^2^College of Life Science, Northeast Agricultural University, Harbin, China; ^3^Lanxi Breeding Farm, Lanxi, China; ^4^Institute of Animal Husbandry, Heilongjiang Academy of Agricultural Sciences, Harbin, China

**Keywords:** gut microbiota, vitamin B1, Min pig, cold adaptation, metagenome

## Abstract

Min pigs exhibit remarkable cold tolerance, where vitamin B1 synthesis by gut microbiota is crucial for the host's energy metabolism. However, the role of this synthesis in cold adaptation of Min pigs are not yet fully understood. This study utilized 16S rRNA amplicon and metagenomic sequencing to examine seasonal variations in the gut microbiota of Min pigs. Results indicated a significant rise in microbial diversity in winter, with the Bacteroidetes group being the most notably increased. The vitamin B1 biosynthetic pathway was significantly enriched during winter, with six significantly upregulated genes (*ThiC, ThiD, ThiE, ThiG, ThiH*, and *ThiL*) showing strong evidence of purifying selection. Among the six vitamin B1 synthesis genes significantly upregulated during winter, the increase was mainly due to a marked elevation in several sequences from specific microbial species. Binding energy analysis revealed that, except for *ThiL*, the average substrate binding energy of the top 10 sequences with the largest seasonal differences was significantly lower than those of the 10 sequences with the smallest differences. Furthermore, most of these sequences were uniquely prevalent in Min pigs and were not found in the homologous sequences of Duroc pigs. Bacteroidetes and Bacteroidales were identified as the primary contributors to these gene sequences. This research provides valuable insights for developing innovative cold-resistant feed and probiotics.

## Graphical Abstract

This study investigated the seasonal effects on the gut microbiota of Min pigs, focusing on Vitamin B1 synthesis. Results revealed a significant increase during winter in Bacteroidota and upregulation of six Vitamin B1 synthesis genes (ThiC, ThiD, ThiE, ThiG, ThiH, ThiL). The ThiE gene had the highest Ka/Ks ratio, indicating potential relaxation of purifying selection. Upregulated sequences showed better substrate binding affinity. 
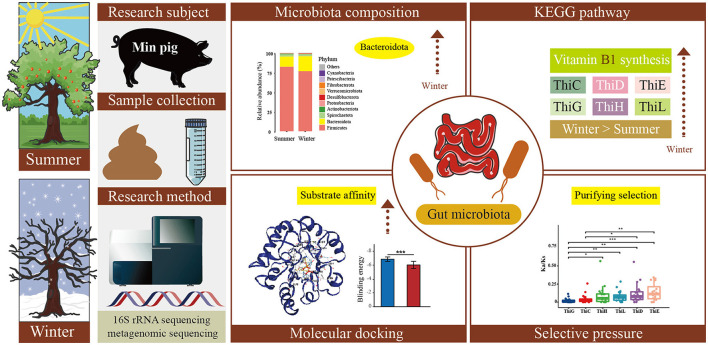

## Introduction

Cold acclimatization confers on organisms the capacity to sustain homeostatic physiological functions under low temperature conditions (Teng et al., 2023). Animals adapt to cold stress mainly through behavioral and physiological changes (Islam et al., 2021). Recent studies have highlighted the crucial role of gut microbiota in the resistance to cold in animals such as mice and pigs (Chevalier et al., 2015; Zhang et al., 2022). Animals inhabiting cold environments undergo adaptive evolution, enhancing their cold tolerance (Wang M. S. et al., 2020). This evolutionary process involves not only changes in the host's genetic regulation, morphological structures, and physiological processes but also extends to alterations in the symbiotic gut microbiota (Patil et al., 2020).

Domestic pigs are one of the most successfully domesticated livestock by humans, providing a rich source of protein. During their long-term domestication, diverse pig breeds have emerged, each with distinct characteristics such as rapid growth, varied meat quality, cold tolerance, and resilience (Zhao et al., 2023). Most breeds, however, are sensitive to low temperatures, leading to increased morbidity, mortality, and significant economic losses in the breeding industry. However, certain pig breeds living in high-altitude or high-latitude regions, such as the Tibetan pig in Tibet and the Min pig in Northeast China, have significantly improved their ability to adapt to extreme cold through long-term natural and artificial selection (Lin et al., 2017). Min pigs from Northeast China exhibit remarkable cold endurance (Teng et al., 2023). Located at China's highest latitude, the Northeast region lies within a mid-altitude area characterized by long and bitterly cold winters. This region is flanked by Eastern Siberia to the north and the Mongolian Plateau to the west, the latter rising over a thousand meters above sea level. Consequently, winter temperatures here are typically lower than in other continental regions at similar latitudes. The Min pig, which has been domesticated in this harsh climate for hundreds of years, has markedly adapted to these extreme conditions. Research indicated that Yorkshire pigs experienced significant damage under chronic cold stress, affecting their small intestines (Sun et al., 2022), lungs (Teng et al., 2022), and hearts (Sun et al., 2023). Conversely, Min pigs showed minimal adverse effects (Teng et al., 2023). Under prolonged low-temperature exposure, Min pigs had a substantially lower mortality rate and reduced incidence of ear tissue frostbite and shivering compared to Large White pigs (Liu et al., 2017). Previous studies have shown that Min pigs and Tibetan pigs shared similar mechanisms for cold resistance (Lin et al., 2017). Therefore, gaining a deeper insight into the biological mechanisms that enable Min pigs to adapt to cold is vital for understanding the cold tolerance of pig breeds in high-altitude regions and for improving the health and productivity of pig farming.

Recent studies have revealed that pigs prioritize glucose for thermogenesis in cold environments (Teng et al., 2023). This process converts sugars into energy, depending on B vitamins, especially vitamin B1 (thiamine). Thiamine serves as a vital coenzyme in several key enzyme complexes, enhancing the effective use of sugar reserves in animals (Hrubsa et al., 2022). Beyond glucose breakdown, cold-adapted pigs, such as Tibetan pigs and Min pigs, generate heat through UCP3-mediated uncoupling in beige adipocytes (Lin et al., 2017). Vitamin B1 is also essential for the thermogenesis in heat-producing adipose tissue (Arianti et al., 2023). Therefore, vitamin B1 is crucial in thermogenic metabolism, assisting organism in maintaining physiological balance and a stable body temperature in cold conditions.

Previous studies have shown that animals lack the ability to independently produce vitamin B1 and therefore rely on dietary sources and gut microbiota for the acquisition of this vital nutrient (Jurgenson et al., 2009; Du et al., 2011; Yoshii et al., 2019; Hrubsa et al., 2022). The genomes of many gut microbiota contain the complete set of genes necessary for vitamin B1 synthesis (Magnúsdóttir et al., 2015; Uebanso et al., 2020). However, some gut microbiota possess only a subset of these genes, suggesting that full vitamin B1 synthesis frequently necessitates inter-species microbial cooperation (Magnúsdóttir et al., 2015; Sharma et al., 2019). Thus, identifying gut microbiota capable of vitamin B1 synthesis could enable targeted probiotic interventions to elevate vitamin B1 levels, thereby boosting host cold resilience. However, previous research primarily focused on the role of metabolites of gut microbiota such as bile acids (Zietak et al., 2016) and short chain fatty acids (Wang D. et al., 2020), neglecting the importance of vitamin B1. The goal of this study was to investigate the influence of the gut microbiota in Min pigs on their capacity to adapt to cold environments by synthesizing vitamin B1 through microbial metabolic processes. We conducted a comparative analysis of the variations in gut microbiota composition and key genes involved in the synthesis of vitamin B1 in Min pigs across two distinct seasons. This research could offer novel breeding strategies for agricultural production, particularly in the livestock industry of colder regions.

## Materials and methods

### Subjects and sample collection

Sample collection was carried out at the Lanxi Min Pig National Conservation Farm, located in Heilongjiang Province, China (126°16'E, 46°15'N). Within the conservation farm, the pig experienced the lowest temperatures during the non-heating period in October. The average highest temperature observed was 12°C, while the average lowest temperature dropped to 0°C. Optimal temperature conditions were recorded in June, characterized by an average yearly maximum of 28°C and a minimum of 17°C. Our experiment subjects was divided into two groups, including a winter group and a summer group. To reduce the potential impact of gender variations, a total of 20 female Min pigs were selected as study subjects. Each pig was 150 days old and weighed ~95 kg. These same 20 female pigs were used for studies in summer and winter. From each pig, a single 200 mg fecal sample was collected per season. Fecal samples were collected in June and October to facilitate a comprehensive analysis of seasonal variations in the gut microbiota. During the experimental period, all growth conditions, including food and water, were kept consistent for the pigs. The collected fecal samples were pooled, with samples from every four individuals combined to create a single composite sample. Pooling is a common method that saves costs while increasing the number of samples analyzed. Since our primary research objective was to identify major shifts in the gut microbiota composition due to seasonal changes, rather than analyzing individual variability, pooling samples was suitable for our research goal. Each group thus produced five pooled samples, which were then subjected to 16S rRNA sequencing. Three samples with the highest consistency from each group were used for metagenomic sequencing based on the 16S rRNA sequencing. The fecal samples were immediately frozen in liquid nitrogen upon collection and subsequently stored at −80°C. The samples were then transported on dry ice to Shanghai Personal Biotechnology Co., Ltd for sequencing.

### 16S rRNA sequencing

Total DNA was extracted from the fecal samples collected from Min pigs (*n* = 10) using the OMEGA Fecal DNA Kit (Omega Bio-Tek, Norcross, GA, USA) according to the manufacturer's protocol. DNA concentration and quality were assessed using a NanoDrop NC2000 spectrophotometer (Thermo Fisher Scientific, Waltham, MA, USA) and agarose gel electrophoresis, respectively. The 338F (55′-ACTCCTACGGGAGGCAGCA-33′) and 806R (55′-GGACTACHVGGGTWTCTAAT-33′) primers were subsequently used to amplify the V3–V4 region of 16S rRNA gene (Caporaso et al., 2011). The PCR conditions consisted of an initial denaturation at 98°C for 5 min followed by 25 cycles of denaturation at 98°C for 30 s, annealing at 50°C for 30 s, extension at 72°C for 45 s, and a final step of 72°C for 5 min. PCR amplicons were purified using Vazyme VAHTSTM DNA Clean Beads (Vazyme, Nanjing, China) and quantified using the Quant-iT PicoGreen dsDNA Assay Kit (Invitrogen, Carlsbad, CA, USA). Pair-end 2 × 250 bp sequencing was performed on the Illumina NovaSeq platform (Illumina, San Diego, CA, USA) according to the manufacturer's instructions.

Raw reads were filtered using FASTP (version 0.18.0) (Chen et al., 2018) to remove low-quality reads. Barcodes and primer sequences were trimmed, and low-quality reads were further filtered out based on the following criteria: sequences with an average Phred score < 20, those containing ambiguous bases, or those with mononucleotide repeats ≥10 bp. Read pairs with a fragment longer than 15 bp and a mismatch error rate of < 1% were then merged using FLASH (v1.2.11) (Magoč and Salzberg, 2011). High-quality sequences were clustered into operational taxonomic units (OTU) at the 97% sequence similarity using UPARSE (version 9.2.64) (Edgar, 2013). Chimeric sequences were removed using UCHIME (v4.2.40) (Edgar et al., 2011) based on the reference database gold database (v20110519). OTU representative sequences were taxonomically identified by alignment against the SILVA_database (version 138) (Quast et al., 2013).

### Shotgun metagenomic sequencing

Metagenomic sequencing libraries were generated with an insert size of 350 base pairs (bp) for six fecal DNA samples following the manufacturer's instructions. The libraries for metagenomic analysis were sequenced on an Illumina NovaSeq platform. The raw reads were treated to remove reads with low qualities, trim the read sequences and remove adaptors using SoapAligner software (Gu et al., 2013). Subsequently, pig genomic DNA sequences were removed by SoapAligner.

Denovo assembly of the high-quality reads was performed using Megahit (v1.2.9) (Li et al., 2016). Scaftigs shorter than 500 bp were removed. The remaining scaftigs were then used to predict open reading frames (ORFs) with MetaGeneMark (v2.10) software (Karl et al., 2022), and sequences shorter than 100 bp were filtered out. CD-HIT software (v4.5.8) (Li and Godzik, 2006) was used to exclude the redundant genes from all predicted ORFs to construct a preliminary nonredundant gene catalog. Subsequently, clean reads of each sample were compared to the preliminary nonredundant gene catalog using Bowtie2 with the default parameters (Langmead and Salzberg, 2012). The number of reads was compared for each gene that could be calculated. The genes with a read number ≤ 2 were removed to obtain a final nonredundant gene catalog. The genes in the final nonredundant gene catalog were called unigenes. The abundance of a gene was calculated based on the number of reads that aligned to the gene, normalizing by the gene length and the total number of reads aligned to the unigenes. Subsequently, we used DIAMOND software (V0.7.9) (Buchfink et al., 2015) to compare the unigenes with the Kyoto Encyclopedia of Genes and Genomes (KEGG) gene database to obtain KO annotation information and metabolic pathway information. We generated raw metagenomic datasets (SRR27494660 to SRR27494665) and amplicon sequencing data (SRR27488203 to SRR27488212) for this study. These datasets are available in the NCBI Sequence Read Archive (SRA) for further research and validation.

### Gut microbiota diversity analysis

Four indices of alpha diversity (i.e., Shannon, Simpson, Chao1 and Richness) were calculated from amplicon sequencing data using the microeco package to assess seasonal variations in microbial diversity between winter and summer (Liu et al., 2021). Furthermore, the hilldiv package (Alberdi and Gilbert, 2019) was employed for the generation of rarefaction extrapolation curves to evaluate gamma diversity of gut microbiota across the winter and summer seasons.

### Microbial community composition analysis

Principal Coordinates Analysis (PCoA) and Venn diagram analyses were performed using the microeco package (Liu et al., 2021) for microbial community structure assessment with amplicon sequencing data. The vegan package was utilized to conduct Analysis of Similarities (ANOSIM) for determining the statistical significance of differences between groups. Welch's *t*-test in the STAMP software (Parks et al., 2014) was applied to analyze seasonal differences in relative abundance of gut microbiota at different taxonomic levels. Lastly, based on the taxonomic profiles of non-redundant genes of metagenomic data, Linear discriminant analysis effect size (LEfSe) was performed to detect differentially abundant taxa across groups using the default parameters.

### Functional profiling of gut microbiota

Functional genes identified through metagenomic sequencing were subjected to KEGG pathway enrichment analysis. KEGG Level 1 pathways were analyzed for seasonal differences using Welch's *t*-test, revealing a significant seasonal variation in “metabolic” pathways, with heightened activity during winter compared to summer. Subsequently, KEGG Level 2 pathways under “metabolic” pathways were selected for differential significance testing. Owing to the extensive number of KEGG Level 3 pathways, the top 30 pathways exhibiting the greatest abundance disparity between seasons were selected for further analysis. Heat maps were generated to visualize these pathways, and random forest analysis was applied to these 30 pathways to identify those most significantly contributing to seasonal differences. Data analysis was conducted using the Majorbio Cloud Platform (https://cloud.majorbio.com/page/tools/) (Ren et al., 2022). For the detection of abundance differences in gut microbial functional genes between seasons, a generalized linear model via edgeR was utilized. Subsequently, genes identified with significantly higher abundance in winter were subjected to KEGG pathway enrichment analysis to elucidate the metabolic pathways in which these genes are involved.

### Bacterial sources and gut microbes involved in vitamin B1 synthesis

Specific gut microbes were identified as the sources for thiamine (vitamin B1) biosynthesis genes. Sankey diagrams were used to visualize the distribution of these genes across their respective gut microbes. Pearson correlation analysis was applied to calculate the correlation between the microbiota and vitamin B1 synthesis genes. Correlation heatmap was presented using Cytoscape V3.9.0 (Shannon et al., 2003) and corrplot V0.84.

### Analysis of selection pressure on vitamin B1 synthetic genes

Duroc pigs, known for their poor cold adaptability, were selected as a control group to analyze the selection pressure on gut microbial functional genes in comparison with Min pigs. Clean reads for six Duroc pig samples [Duroc-JY2 (CNS0178532), Duroc-JY3 (CNS0178533), Duroc-JY10 (CNS0178540), Duroc-JY13 (CNS0178543), Duroc-JY15 (CNS0178545), and Duroc-JY16 (CNS0178546)] were obtained from the National Genomic Data Center (CNGBdb) Life Big Data Platform (Chen et al., 2021). CDS sequences from Min pigs' metagenomic sequencing and the downloaded CDS sequences from the Duroc samples served as databases for pairwise sequence alignment of orthologs. Alignments with coverage and identity exceeding 80% were identified using NCBI BLAST 2.11.0, with parameters set to -e 1e-5. Homologous gene pairs between the two datasets, totaling 161,858 pairs, were determined by intersecting these alignments. Selection pressure on these gene pairs was analyzed using the Simple Ka/Ks Calculator plugin in TBtools (Chen et al., 2020), which facilitated the calculation of nonsynonymous (Ka) and synonymous (Ks) substitution rates, and their ratio (Ka/Ks).

### Protein structure prediction and molecular docking

For each of the six vitamin B1 synthetic genes upregulated in winter, the top 10 sequences with the largest differential abundance between winter and summer, and the bottom 10 sequences with the least differential abundance were selected. These sequences were used for protein structure prediction and molecular docking analysis. The aim was to predict the binding energy between these enzymes and their substrates, and to assess whether sequences with significant winter abundance increase demonstrated enhanced substrate binding capabilities compared to those exhibiting minimal seasonal variation. Protein 3D structures were predicted using ColabFold v1.5.3 (Mirdita et al., 2022). Subsequent molecular docking and binding energy calculations were performed through structure-based blind docking with CB-Dock2 (Liu et al., 2022). The substrate ligand files in SDF format (C00082, C04556, C04752, C04327, C20247, C03373, C15809, C11437, C01081) for molecular docking were sourced from the PubChem database. Visualization of the protein 3D structures was conducted using the EzMol 2.1 website (Reynolds et al., 2018). For the six vitamin B1 synthesis genes, phylogenetic trees were constructed individually for each gene using all available sequences in the MEGA 11 software. Multiple sequence alignment was performed with Muscle, followed by sequence trimming using triAI. Optimal models for the genes were then determined. Phylogenetic analysis employed the Maximum Likelihood (ML) method, utilizing a Bootstrap value of 1,000. These trees were based on the coding sequences (CDS) from metagenome of Min pigs, specifically focusing on *ThiC, ThiD, ThiE, ThiG, ThiH*, and *ThiL* genes in the gut microbiome.

## Results

### Changes in gut microbiome diversity in cold environments

**Figure 1:**
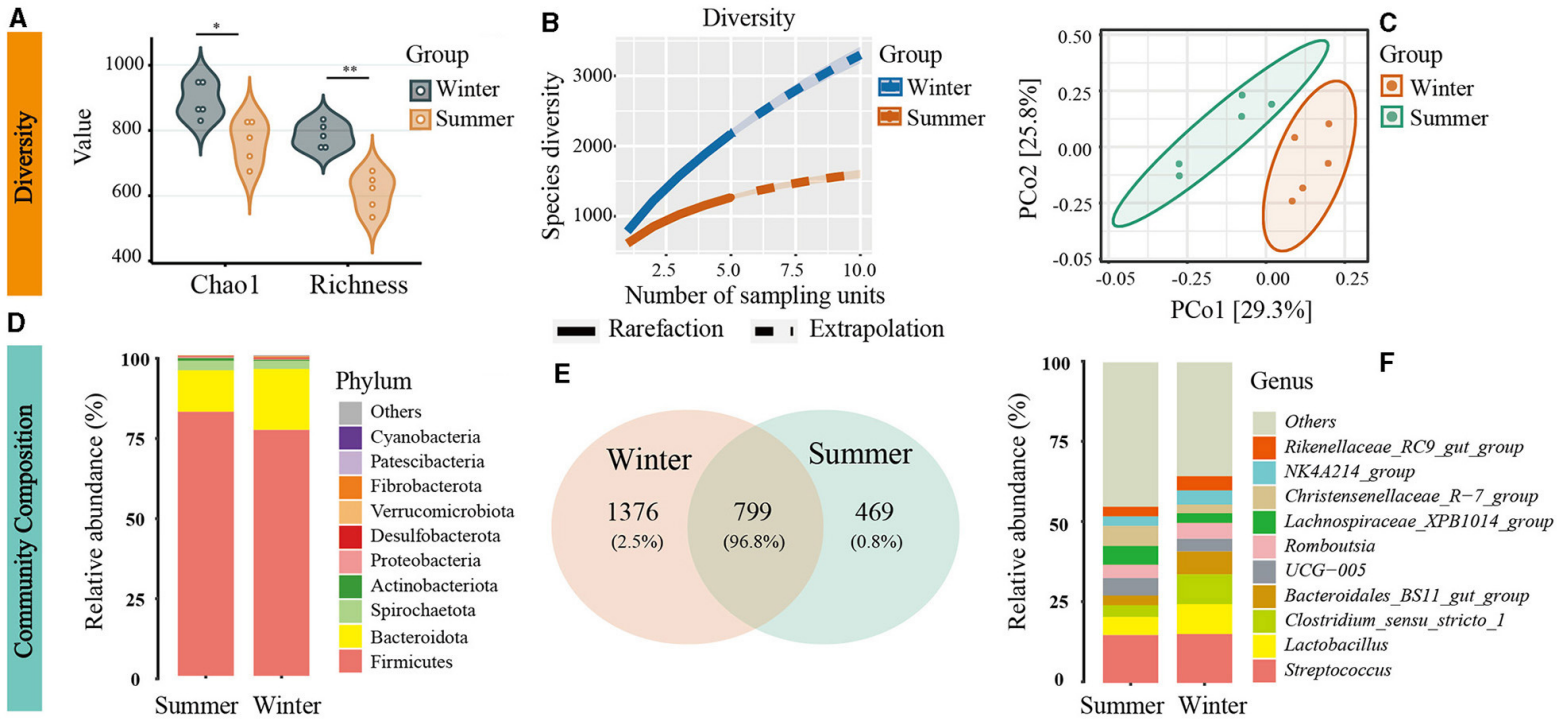
Seasonal variation in gut microbiota diversity and composition in Min Pigs. **(A)** Alpha diversity with significance denoted by asterisks (*P < 0.05, **P < 0.01). **(B)** Gamma diversity assessed using neutral Hill numbers (*q* = 1) with rarefaction (solid line) and extrapolation (dashed line) curves. **(C)** Beta diversity illustrated by Principal Coordinates Analysis (PCoA) plots, highlighting distinct microbial communities in winter and summer. **(D)** Comparison of the top 10 gut microbiota at the phylum level based on relative sequence abundance. **(E)** Venn diagram of operational taxonomic units (OTUs) showing shared and unique microbial species between winter and summer. **(F)** Comparison of the top 10 gut microbiota at the genus level based on relative sequence abundance.

In the analysis of 10 samples using amplicon sequencing, sequence counts varied between 18,087 and 35,274. To standardize for comparability, all samples were rarefied to 18,087 sequences. A total of 2,644 OTUs were identified, including 22 phyla. A significant increase in gut microbial diversity was observed during winter (Figures 1A, B). There were 1,376 OTUs unique to winter, in contrast to 469 unique OTUs in summer, indicating a substantially higher number of unique OTUs in the winter than in summer (Figure 1E). Diversity analysis showed that the α diversity indices were significantly higher in winter than in summer (Chao1: *t* = −3.29, *P* = 0.01; Richness: *t* = −5.65, *P* = 0.001, Figure 1A). Rarefaction-extrapolation curves also demonstrated a higher γ diversity of the gut microbiota during winter (Figure 1B).

### Changes in the gut microbial composition in cold environments

**Figure 2:**
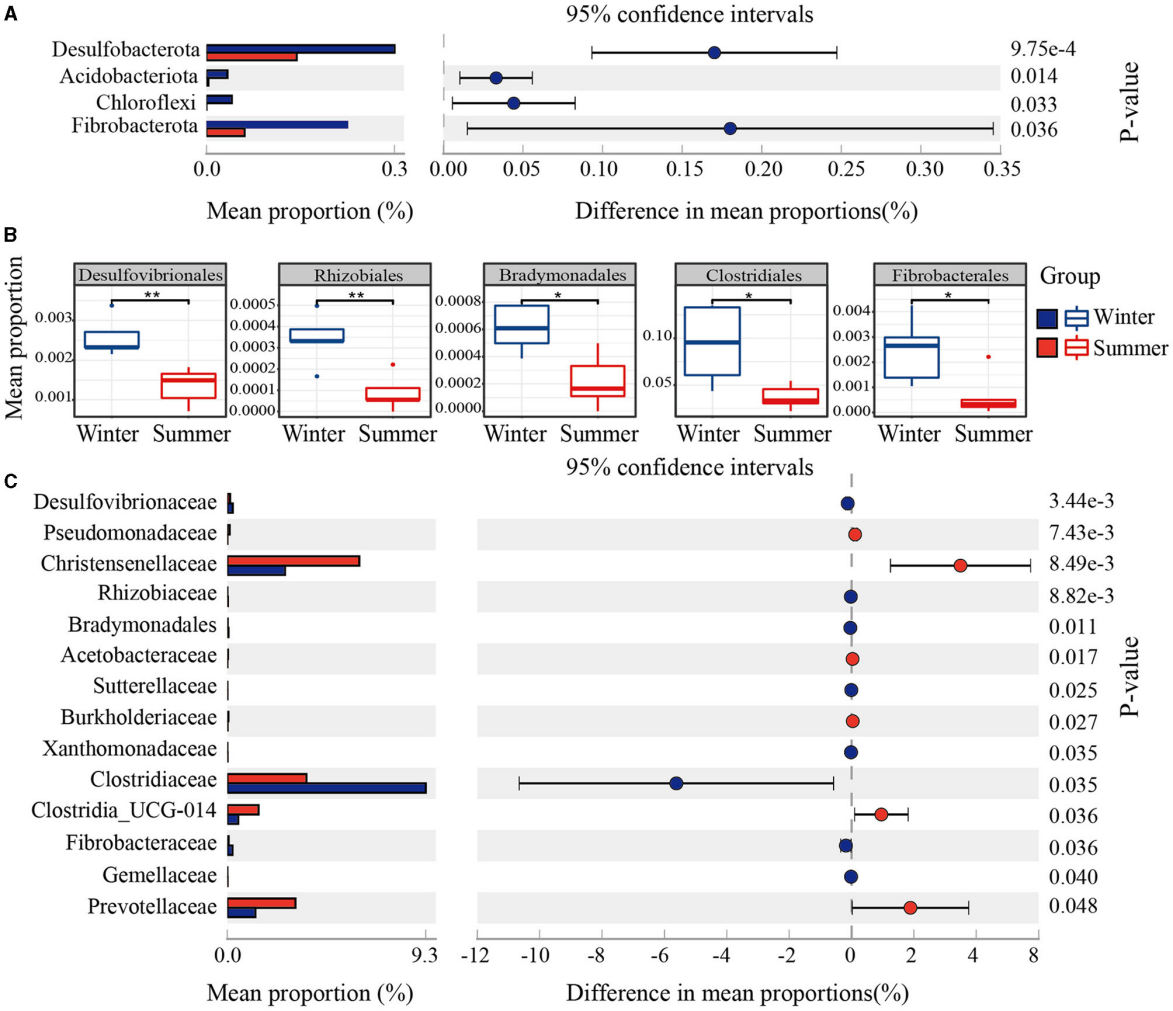
Seasonal variation in gut microbial composition. **(A)** Phylum-level mean proportions (%) in winter and summer. **(B)** Order-level variations shown in boxplots. **(C)** Family-level mean proportions (%) across seasons, indicating significant variation with 95% CIs and *P*-values.

PCoA and ANOSIM revealed significant seasonal differences in the species composition of the gut microbiota (*r* = 0.52, *P* = 0.006; Figure 1C). Firmicutes and Bacteroidetes were the predominant bacterial phyla in the Min pigs' gut microbiota across both seasons. The abundance of Firmicutes sequences decreased from 82% in summer to 77% in winter, while Bacteroidetes increased from 13% to 19% over the same period (Figure 1D). Using amplicon data, a marked increase in the *Bacteroidales_BS11_gut_group* at the genus level was observed during winter, as depicted in Figure 1F. Furthermore, LEfSe analysis of metagenomic data revealed a significant increase in the abundance of the Bacteroidetes phylum and Bacteroidales order during winter, establishing them as the dominant microbiota with the most pronounced seasonal fluctuations (Supplementary Figure S1). Beyond Bacteroidetes, we used amplicon data to analyze seasonal variations in microbial community composition at different taxonomic levels. We observed significant increases in various taxa within the gut microbiome during winter, including four phyla (Desulfobacterota, Acidobacteriota, Chloroflexi, Fibrobacterota), 11 orders (Desulfovibrionales, Rhizobiales, Bradymonadales, Clostridiales, Fibrobacterales, etc.), and 14 families (Desulfovibrionaceae, Rhizobiaceae, Bradymonadaceae, Sutterellaceae, Xanthobacteraceae, Clostridiaceae, Fibrobacteraceae, Gemellaceae, etc.), as shown in Figures 2A–C and Supplementary Table S1. At the genus level, 30 genera, including *Clostridium sensu stricto 1, Roseburia*, and *Fibrobacter*, and so on, also exhibited notable increases during the winter (see Figure 1F and Supplementary Table S1). At the OTU level, the most significant seasonal abundance differences were observed in *Clostridium sensu stricto 1* and *Bacteroidales BS11 gut group* (Supplementary Table S1).

### Changes in gut microbiota functional profiling and enrichment of vitamin B1 metabolic pathways in cold environments

**Figure 3:**
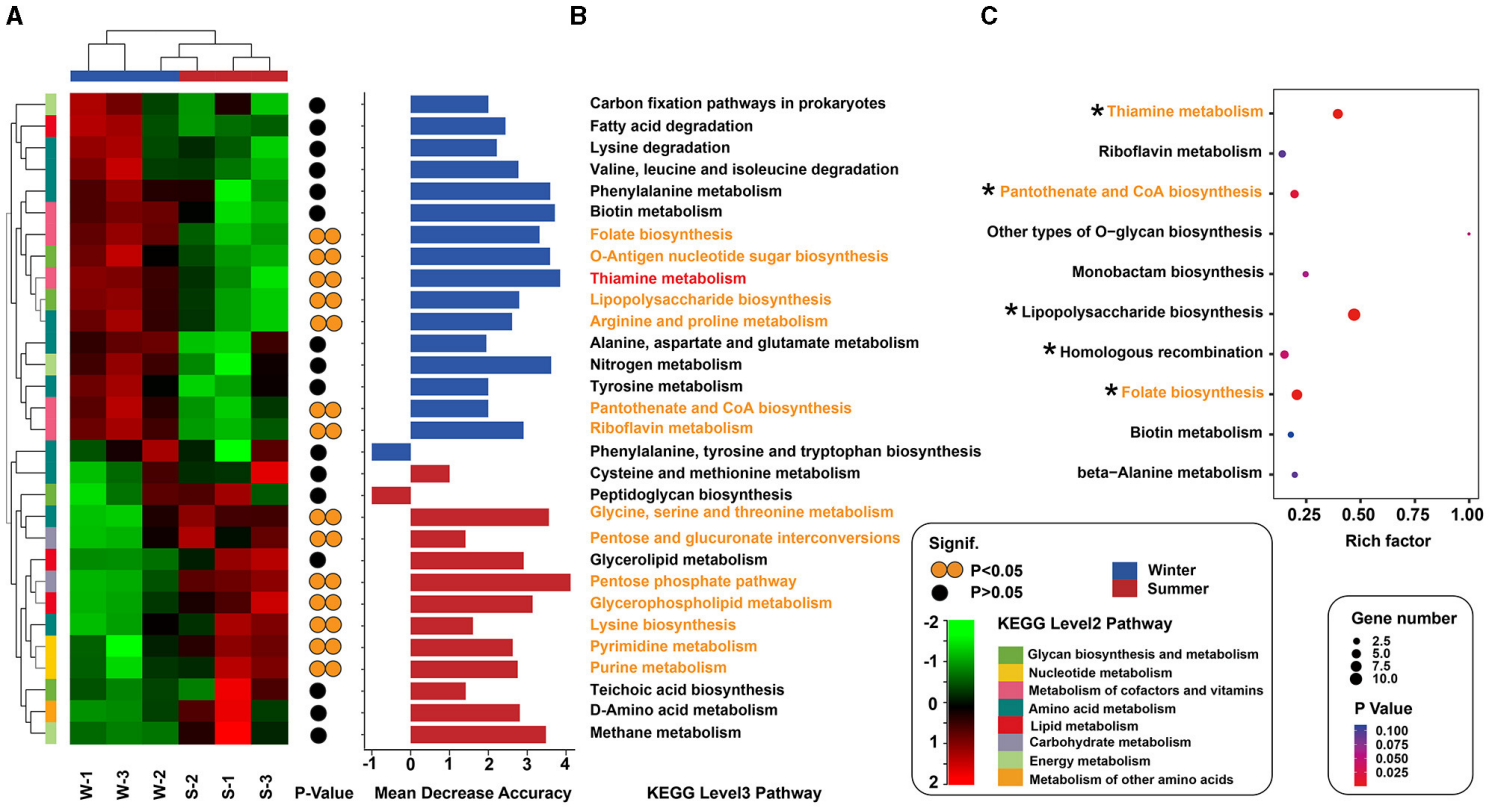
Seasonal changes in metabolic pathways of gut microbiota. **(A)** Heatmap shows the 30 pathways with the most significant seasonal variation, colored from red (high abundance) to green (low abundance). Yellow circles indicate statistical significance (*P < 0.05). **(B)** Importance of each pathway is determined by random forest analysis, with blue for winter and red for summer. **(C)** Bubble plot displays the top 10 pathways enriched for genes upregulated in winter, with rich factor (proportion of upregulated genes) and bubble size for gene count, and color for *P*-value significance. Asterisks highlight pathways with significant differences (*P < 0.05).

Metagenomic sequencing identified 1,983,499 genes in Min pigs' gut microbiota, suggesting notable seasonal variations in functional profiles. Specifically, 22,845 genes showed significant variation (*P* < 0.001), with 4,027 unique to summer and 4,877 to winter (Supplementary Figure S2). PCoA further confirmed these seasonal differences in functional profiles (Supplementary Figure S3). Using Welch's *t*-test to assess differences in KEGG Level 1 pathways, we found a significant increase in the “Metabolism” category in the winter season compared to the summer period (*P* < 0.05, Supplementary Figure S4A). Further analysis at KEGG Level 2 showed a significant rise in the “Metabolism of cofactors and vitamins” pathway during winter (*P* < 0.05, Supplementary Figure S4B). In a detailed analysis of the “Metabolism” category, we ranked the seasonal differences in KEGG Level 3 pathways by absolute values and focused on the top 30 with the most significant variations. This analysis identified seven pathways particularly enriched in winter, including Thiamine, O-Antigen nucleotide sugar, Arginine and Proline, Folate, Lipopolysaccharide, Pantothenate and CoA, and Riboflavin metabolism. These pathways were chiefly involved in the metabolism of B-group vitamins (B1, B2, B5, and B9), as shown in Figure 3A and Supplementary Table S2.

**Figure 4:**
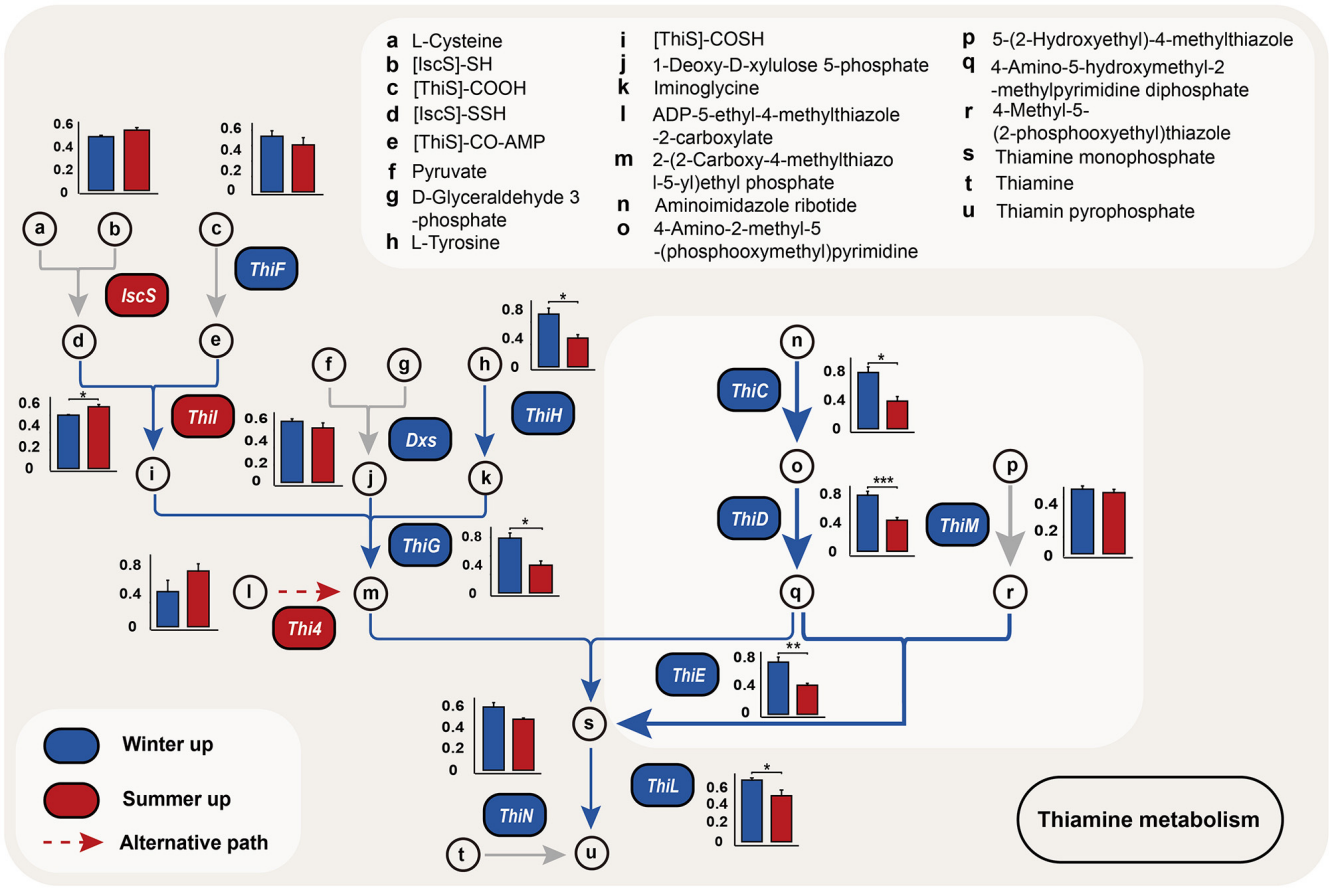
Seasonal variation in thiamine biosynthesis genes. This figure compares the abundance of genes involved in the thiamine biosynthesis pathway within microbial communities, using metagenomic data from winter (blue) and summer (red). The bars, marked a-u, show the genes involved in forming thiamine and their derivatives. Dashed lines suggest an alternative biosynthetic pathway. Asterisks mark statistically significant differences in gene abundance (*P < 0.05, **P < 0.01, ***P < 0.001).

Random forest analysis revealed that, among the metabolic pathways with significant differences, the thiamine metabolism pathway exhibited the highest contribution to seasonal variations in Min pigs (Figure 3B). We also identified genes with differential abundance between winter and summer. KEGG pathway analysis indicated that, among the top 10 enriched pathways for genes upregulated in winter, five were related to B-group vitamin metabolism, notably vitamin B1, B2, B5, B9, and B7, with B1 being the most prominently represented (Figure 3C, Supplementary Table S3). In the vitamin B1 metabolic pathway, 10 out of 13 synthesis-related genes were more abundant in winter, with six (*ThiC, ThiD, ThiE, ThiG, ThiH*, and *ThiL*) showing significant differences (*P* < 0.05, Figure 4, Supplementary Table S4).

### Analysis of selective pressure on vitamin B1 biosynthesis genes

**Figure 5:**
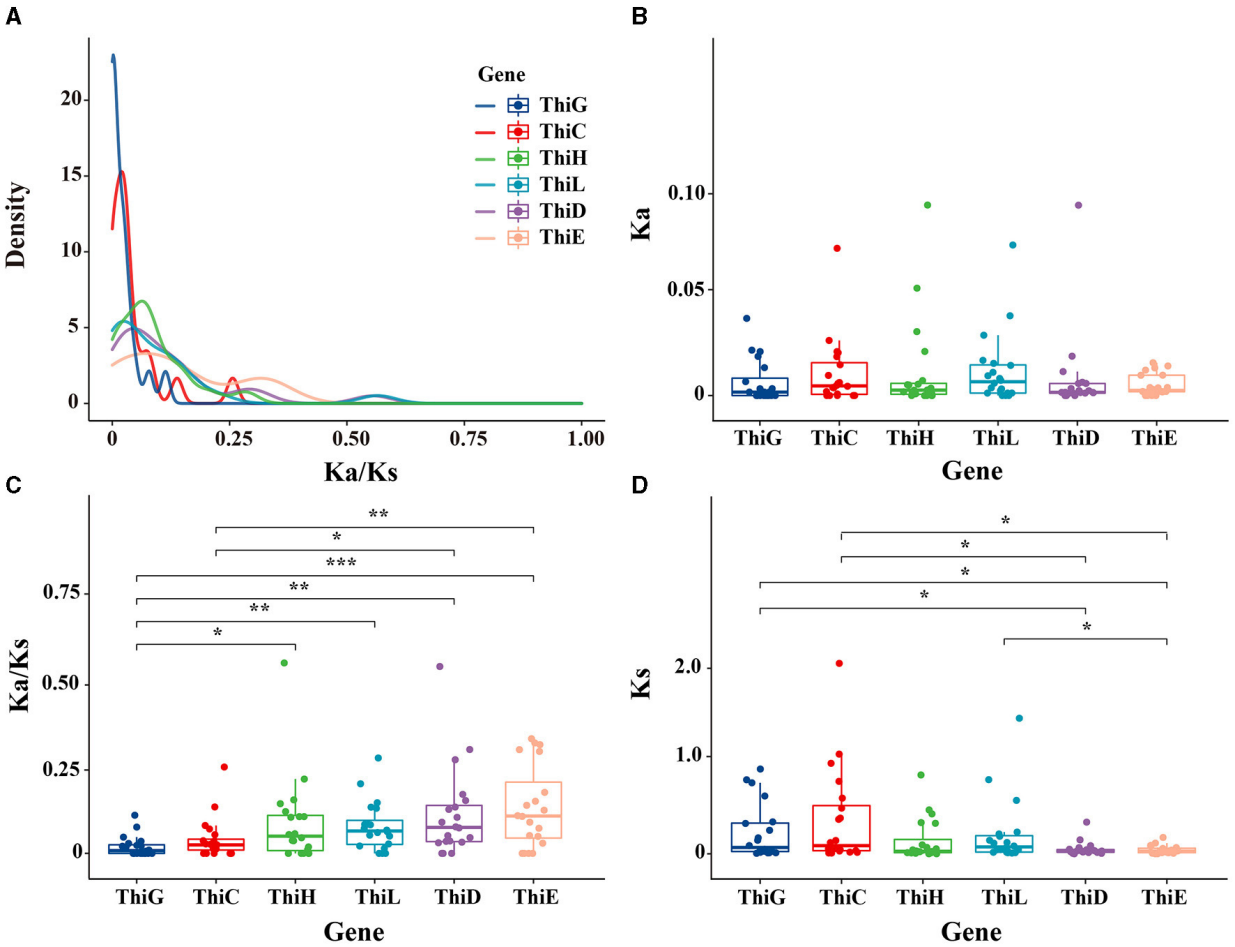
Analysis of selective pressure on vitamin B1 biosynthesis genes. **(A)** The distribution of nonsynonymous to synonymous substitution ratios (Ka/Ks). **(B)** The nonsynonymous substitution rate (Ka) per gene, with each dot representing an individual comparison. **(C)** The Ka/Ks ratio variations across the genes. **(D)** The synonymous substitution rates (Ks). Asterisks mark statistically significant differences (*P < 0.05, **P < 0.01, ***P < 0.001).

We screened orthologous genes between Min and Duroc pigs to calculate their nonsynonymous (Ka) to synonymous (Ks) substitution rates. Specifically, for the winter, we identified six vitamin B1 synthesis genes significantly upregulated and ranked their sequences by relative abundance. From each gene, the top 20 sequences were selected to calculate their mean Ka/Ks ratio, as detailed in Supplementary Table S5. The average Ka/Ks ratio for these sequences across all genes was below 1, suggesting strong purifying selection. Remarkably, *ThiE* was observed to have the highest Ka/Ks ratio, with an average value of 0.14. This ratio was significantly higher than that of *ThiC* (*P* < 0.01) and *ThiG* (*P* = 0.00044; Figures 5A, C). Despite the lack of significant differences in the Ka values among the six genes (Figure 5B), the Ks value for *ThiE* was substantially lower compared to both *ThiC* and *ThiG* (*P* < 0.05 for both). This pattern indicated that the elevated Ka/Ks ratio in *ThiE* was predominantly attributed to a reduced rate of synonymous mutations, as shown in Figures 5C, D. Such findings suggested a potential relaxation of purifying selection in *ThiE* during the evolutionary domestication process of Min pigs, compared to earlier stages.

### Protein modeling and molecular docking for vitamin B1 biosynthesis genes

**Figure 6:**
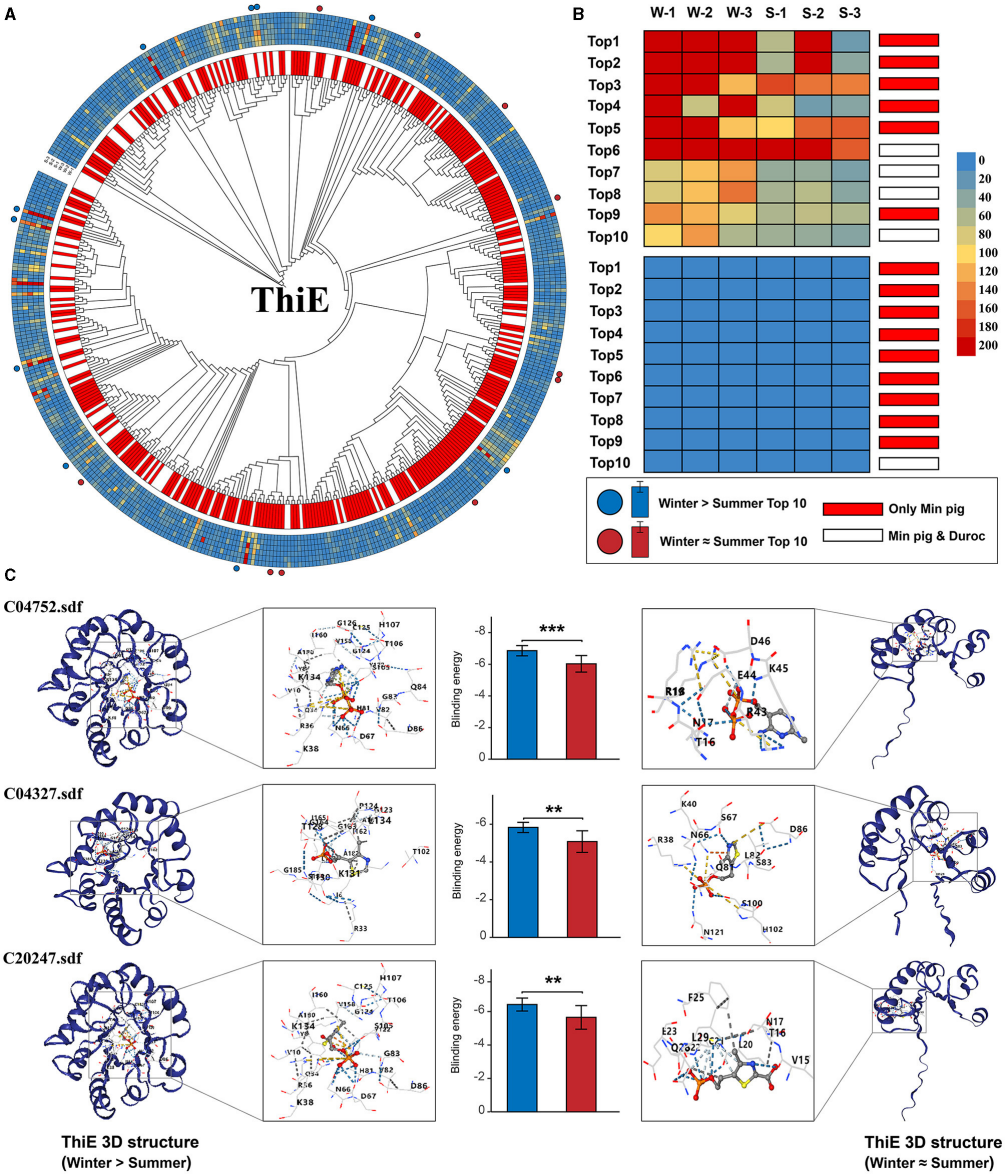
Seasonal variation in *ThiE* gene sequences and protein binding efficiency. **(A)** Phylogenetic tree of *ThiE* sequences with orthologous gene pairs between Min pigs and Duroc pigs indicated in the inner circle. Unique sequences to Min pigs are marked in red, shared sequences in white. Sequence abundance across samples is represented in the outer circle, with the top 10 sequences with the largest (red dot) and smallest (blue dot) winter-summer abundance disparities highlighted in the outermost bands. **(B)** The top 10 sequences with the largest and smallest seasonal abundance changes, showing their abundance in winter (W-1, W-2, W-3) and summer (S-1, S-2, S-3) samples. **(C)** Structural models and molecular docking simulations of the top 10 sequences with the largest and smallest seasonal variation. On the left side, proteins with the highest binding affinities are shown. On the right side, proteins with the lowest affinities are displayed. Asterisks mark statistically significant differences (**P < 0.01, ***P < 0.001).

We analyzed the abundance of each gene's sequences across winter and summer and calculated the difference in sequence counts between these seasons. The sequences were then ranked based on the magnitude of their seasonal variation. Our analysis revealed that the sequences with the largest difference in abundance between winter and summer also exhibited the highest total abundance across both seasons. The notable increase in gene abundance during winter was primarily attributable to a few sequences that exhibited the greatest seasonal variation. *ThiE* stood out among the six genes due to its unique selection pressure results, highest sequence diversity, and the broadest range of source microorganisms, as shown in Supplementary Table S6. We therefore chose *ThiE* for molecular docking simulations to estimate the binding energies with its three substrates, focusing on sequences with the most pronounced seasonal variations. This simulation aimed to predict the binding energy of the ThiE protein with its three substrates. We specifically selected the top 10 *ThiE* sequences exhibiting the largest and smallest differences in winter-summer abundance for this analysis. Molecular docking was performed for each of these sequences against the three substrates. Our findings, as detailed in Figure 6C and Supplementary Table S7, revealed that the average binding energy of the 10 sequences with the greatest winter-summer differences was significantly lower compared to those with the smallest differences. This pattern, except for *ThiL* which exhibited no significant variation, was consistent across the remaining five genes (Supplementary Figures S5–S9, Supplementary Table S7). Significantly, among the top 10 *ThiE* sequences exhibiting the largest seasonal variations, the majority were exclusive to Min pigs. Specifically, for these sequences, corresponding homologous sequences were not identified in the gut metagenomic datasets of Duroc pigs (six out of 10 sequences; Figures 6A, B). Moreover, for each of the other five genes studied, the sequence displaying the greatest difference in abundance between winter and summer was also uniquely found in Min pigs, indicating a similar trend of species-specific variation.

### Characterization of gut microbiota involved in vitamin B1 synthesis

**Figure 7:**
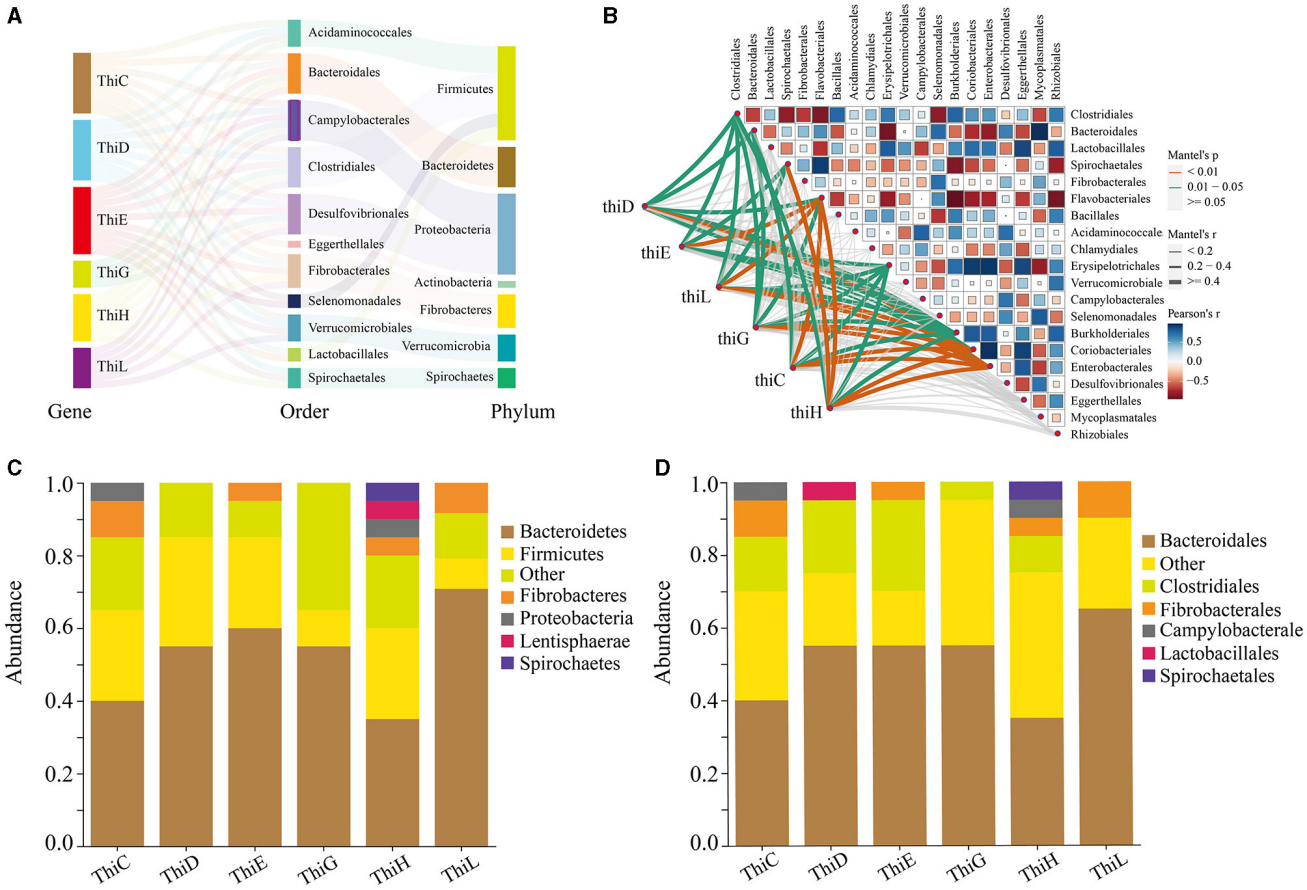
Sources of vitamin B1 biosynthesis genes in gut microbiota. **(A)** A Sankey diagram showing the microbial sources of six genes involved in vitamin B1 synthesis. **(B)** A correlation network displaying the relationship between these genes and the 20 most abundant bacterial orders. The heatmap illustrates the correlation strength (Pearson's *r*), and the lines represent significance according to Mantel's test. **(C)** Phylum-level source bacteria proportions for the 20 sequences with the largest abundance fluctuations between winter and summer for these genes. **(D)** Order-level source bacteria proportions for these sequences.

In our study, we found that among the six vitamin B1 synthesis genes upregulated in winter, sequences for five of these genes (*ThiC, ThiD, ThiE, ThiG*, and *ThiL*, excluding *ThiH*) showed the most significant seasonal fluctuations in abundance and originated from the phylum Bacteroidetes (Figure 7A, Supplementary Table S6). Analysis of the top 20 sequences with the greatest seasonal differences across all six genes revealed a significant presence from both Bacteroidetes and Bacteroidales, with Bacteroidetes representing 75% in *ThiL*, 60% in *ThiE*, and 55% each in *ThiD* and *ThiG* (Figure 7C). This trend was consistent for Bacteroidales (Figure 7D), underscoring their crucial role in vitamin B1 synthesis in Min pigs. Moreover, microbial sources for these genes extended to species from the phyla Firmicutes, Fibrobacteres, Proteobacteria, Actinobacteria, Verrucomicrobia, and Spirochaetes, and orders including Bacteroidales, Clostridiales, Desulfovibrionales, and others contributing to vitamin B1 synthesis (Figure 7A, Supplementary Table S8).

Correlation analysis was conducted between the top 20 bacterial orders in abundance and the six vitamin B1 synthesis genes. The results indicated that the abundance of all six genes was significantly positively correlated with the abundance of Flavobacteriales, Burkholderiales, Coriobacteriales, and Enterobacterales. Five genes exhibited a significant positive correlation with the abundance of Erysipelotrichales, four genes were positively correlated with Clostridiales, and three genes showed a significant positive correlation with Spirochaetales (Figure 7B, Supplementary Table S9). These findings suggested that these intestinal microbes may collaborate synergistically in the biosynthesis of vitamin B1.

## Discussion

Previous research showed that Min pigs adapted to low temperatures primarily through enhanced glucose metabolism for thermogenesis (Teng et al., 2023). Compared to Yorkshire pigs, Min pigs demonstrated superior cold adaptability, attributed to their more efficient glucose absorption, gluconeogenesis, and overall metabolic efficiency during cold exposure (Teng et al., 2023). Glucose metabolism relied on vitamin B1 (thiamine) as an essential coenzyme. This vitamin was exclusively obtainable through dietary sources or gut microbiota biosynthesis, highlighting the critical role of gut microbiota. Our research uncovered a notable winter-specific enhancement in thiamine biosynthetic pathways within the gut microbiota of Min pigs. This finding suggested an elevated microbial synthesis of thiamine during colder months. Thiamine pyrophosphate (TPP), the active form of thiamine, was particularly vital for the pyruvate dehydrogenase complex (PDHC), α-ketoglutarate dehydrogenase complex (KGDHC), and transketolase (TK) reactions. These enzymes were central to effective glucose utilization for ATP production and facilitated the gluconeogenic pathway (Lonsdale, 2006). Beyond glucose metabolism, fat metabolism serves as a crucial pathway for thermogenesis, playing a key role in heat production and energy metabolism in animals. A study by Arianti et al. (2023) highlighted the critical role of thiamine in activating thermogenic functions in human brown and beige adipocytes. They found that, in the human neck region, adipocytes under cAMP induction express high levels of the thiamine transporter ThTr2, leading to substantial thiamine consumption. Inhibition of ThTr2 resulted in reduced thiamine uptake and decreased thermogenic activity in these cells. Furthermore, thiamine supplementation was shown to enhance the expression of genes associated with adipocyte thermogenesis (Arianti et al., 2023). These findings underscore the importance of adequate thiamine levels for the activation of thermogenic functions in human adipocytes. In this context, gut microbiota synthesis of vitamin B1 in Min pigs plays a pivotal role in supporting host glycolysis and fat-induced thermogenesis, thereby contributing significantly to the pigs'cold adaptability.

Our study identified significant upregulation of six key genes in the vitamin B1 synthesis pathway during winter, namely *ThiC, ThiD, ThiE, ThiG, ThiH*, and *ThiL*. Thiamine synthesis comprises two distinct parts: the formation of thiazole and pyrimidine moieties. For the pyrimidine moiety, ThiC converts 5-aminoimidazole ribonucleotide (AIR) into 4-amino-5-hydroxymethyl-2-methylpyrimidine phosphate (HMP-P), which is then phosphorylated by ThiD to HMP-PP (Jurgenson et al., 2009). Thiazole moiety synthesis involves several steps: 1-deoxy-D-xylulose 5-phosphate synthase (Dxs) synthesizes 1-deoxy-D-xylulose 5-phosphate (DXP) from glyceraldehyde 3-phosphate and pyruvate; ThiF adenylates the sulfur carrier protein ThiS, producing a thiocarboxylate; ThiO or ThiH converts glycine or tyrosine into dehydroglycine, which then reacts with DXP and ThiS's thiocarboxylate under ThiG's catalysis to form an isomeric thiazole phosphate carboxylate. Subsequently, TenI aromatizes it to form thiazole phosphate carboxylate ester, and finally, ThiE couples THZ-P with HMP-PP to form thiamine monophosphate (ThMP). Finally, ThiL phosphorylates ThMP to the active Thiamine diphosphate (ThDP) (Jurgenson et al., 2009). These enzymatic steps, significantly enhanced in winter, are crucial in the thiamine biosynthesis process.

Metagenomic sequencing, involving multiple microorganisms, revealed that each gene's sequence consisted of various heterogeneous sequences from different microbes, showing significant homologous variation. Selection pressure analysis showed that for the top 20 most abundant sequences of each gene, the average Ka/Ks ratio was significantly below 1, indicating strong purifying selection. However, the higher Ka/Ks ratio of *ThiE*, mainly due to its lower Ks value, suggested a relaxation in purifying selection during the domestication of Min pigs. We hypothesized that in the prolonged cold environment of Northeast China, these vitamin B1 synthesis genes had undergone intense purifying selection. *ThiE*, owing to its critical role, might have adapted to the local conditions earlier, leading to a subsequent relaxation in selection pressure. Notably, *ThiE* also displayed the highest sequence diversity among the six genes, reflecting its origin from a diverse range of microbial species. This finding, along with the distinct selection pressure outcomes for *ThiE*, led us to focus our in-depth investigation on its sequence variations. In our study, we observed a significant winter-specific increase in the abundance of certain sequences within the *ThiE* gene, compared to summer. Notably, the most increased sequences were also the most abundant overall, suggesting their heightened importance in winter. Similar trends were noted in the analysis of the other five genes. Molecular docking simulations revealed that these abundant *ThiE* sequences exhibited enhanced binding affinity to their substrates, indicating increased enzymatic activity during winter. This finding implied that Min pigs adapted to cold environments may have more efficient vitamin B1 synthesis genotypes and gut microbiota, facilitating a more effective synthesis of substantial amounts of vitamin B1 in the winter season.

We analyzed the bacterial origins of the sequences with the greatest seasonal variations in abundance for six vitamin B1 synthesis genes. The results showed that the sequences of *ThiC, ThiD, ThiE, ThiG*, and *ThiL* with the most significant seasonal variations predominantly originated from the phylum Bacteroidetes. Except for *ThiL*, the sequences of the remaining five genes were all classified under the order Bacteroidales. We found a pronounced winter increase in both the Bacteroidetes phylum and the Bacteroidales order, marking them as the most significantly elevated taxa in the Min pigs' winter gut microbiome. This elevation suggested that they were distinctive markers of the winter gut microbiota and underscores the role of the gut microbiota in enhancing vitamin B1 synthesis during winter in Min pigs. Magnúsdóttir et al. (2015) conducted a bioinformatics-based systematic genomic assessment of various gut microbes, identifying Bacteroidetes and Fusobacteria as principal phyla in thiamine pyrophosphate (TPP) synthesis (Magnúsdóttir et al., 2015). Similarly, Pan et al. (2017) reported a positive correlation between thiamine concentration in bovine rumen and Bacteroidetes abundance. Therefore, our research findings were consistent with both bioinformatics analysis and experimental data. In summary, the Bacteroidetes phylum and the Bacteroidales order play a key role in synthesizing vitamin B1, offering both theoretical and practical benefits for health management and dietary supplementation. In probiotics research, exploiting Bacteroidetes and Bacteroidales for vitamin B1 synthesis opens avenues for crafting more effective probiotic formulas. Such innovations could enhance vitamin B1 production, improving overall health. This strategy is particularly vital for those at risk of vitamin B1 deficiency due to dietary imbalances or specific health conditions.

We observed a significant enhancement in the metabolic pathways of other B-group vitamins, including B2, B5, B7, and B9, during winter. This suggests a synergistic and interdependent role of these vitamins in substance metabolism and energy generation. In alignment with our results, Park et al. (2022) reported that *Faecalibacterium prausnitzii* converted pyruvate to acetyl-CoA using pyruvate:ferredoxin oxidoreductase during butyrate synthesis, with vitamins B1 and B2 serving as essential coenzymes, directly influencing butyrate production. This role of B2 was further confirmed by Liu et al. (2023), who found that vitamin B2 supplementation in healthy mice significantly increased the abundance of *Faecalibacterium prausnitzii* and butyrate production. Additionally, Wan et al. (2022) demonstrated the involvement of vitamins B1 and B5 in the tricarboxylic acid cycle, crucial for energy release and thermoregulation. These findings highlight the collective role of multiple B vitamins in thermogenesis and energy metabolism (Kurowska et al., 2023).

The potential of B-vitamin supplementation to enhance cold tolerance in domestic animals and humans represents a fascinating field of study. This extends beyond theoretical research, as its practical implications in formulating human nutritional supplements and animal feed are significant. However, the current development of probiotic resources is relatively delayed, and the discovery of novel probiotic strains remains insufficient, thereby constraining the healthy growth of related industries. Our results indicated that various specific microbes within the phylum Bacteroidetes and the order Bacteroidales can served as excellent candidates for developing a new generation of probiotics aimed at enhancing host cold tolerance. Additionally, it is proposed that using a combination of multiple B vitamins could enhance the host's cold tolerance more effectively.

Our research underscored the ecological significance of understanding animal survival in harsh, cold environments, such as those at high altitudes and latitudes. High-altitude areas presented unique challenges to livestock due to decreased oxygen levels and lower temperatures, which significantly impacted metabolic pathways and microbial diversity. Our findings elucidated potential adaptive strategies that Min pigs might have employed to mitigate cold stress. Notably, the upregulation of thiamine synthesis pathways during the winter months enhanced metabolic efficiency and thermogenesis, which was crucial for surviving in the extreme cold of high-altitude and latitude environments. This adaptability was crucial not only for effective livestock management and breeding strategies in these regions but also highlighted the challenges traditional livestock breeds faced. Consequently, our study deepened the understanding of adaptability within the gut microbiota and emphasized the potential to develop probiotics or nutritional interventions that could bolster livestock resilience under the severe conditions prevalent at high altitudes and latitudes.

## Data availability statement

The datasets presented in this study can be found in online repositories. The names of the repository and accession numbers can be found in the article.

## Ethics statement

The animal study was approved by Institutional Animal Care and Use Committee of Northeast Agricultural University. The study was conducted in accordance with the local legislation and institutional requirements.

## Author contributions

YC: Conceptualization, Data curation, Funding acquisition, Methodology, Software, Validation, Visualization, Writing – original draft, Writing – review & editing. ZZ: Formal analysis, Software, Validation, Visualization, Writing – review & editing. JC: Investigation, Resources, Writing – review & editing. CW: Investigation, Resources, Writing – review & editing. DL: Writing – review & editing. ZL: Writing – review & editing. CX: Funding acquisition, Project administration, Supervision, Visualization, Writing – original draft, Writing – review & editing.
