# Supplementary material for: Coevolution of specific gut microbiota of Min pig with host cold adaptation through enhanced vitamin B1 synthesis
Source: Front Microbiol. 2024 Aug 30;15:1448090. doi: 10.3389/fmicb.2024.1448090 (PMC11401075; doi:10.3389/fmicb.2024.1448090)
Supplement: Supplementary file 1 [file Data_Sheet_1.docx]

Supplementary Material

**Coevolution of Specific Gut Microbiota with Host Cold Adaptation Through Enhanced Vitamin B1 Synthesis**

Yang Chang^1,2^,†, Ziwen Zhang^1,2^,†, Jiancheng Cai^3^, Chunan Wang^3^,

Di Liu^4^, Zhonghua Liu^1,2,*^, Chunzhu Xu^1,2,*^

**Table of Contents:**

| **Supplementary Figure S1** | Page 2 |
| --- | --- |
| **Supplementary Figure S2** | Page 3 |
| **Supplementary Figure S3** | Page 4 |
| **Supplementary Figure S4** | Page 5 |
| **Supplementary Figure S5** | Page 6 |
| **Supplementary Figure S6** | Page 7 |
| **Supplementary Figure S7** | Page 8 |
| **Supplementary Figure S8** | Page 9 |
| **Supplementary Figure S9** | Page 10 |


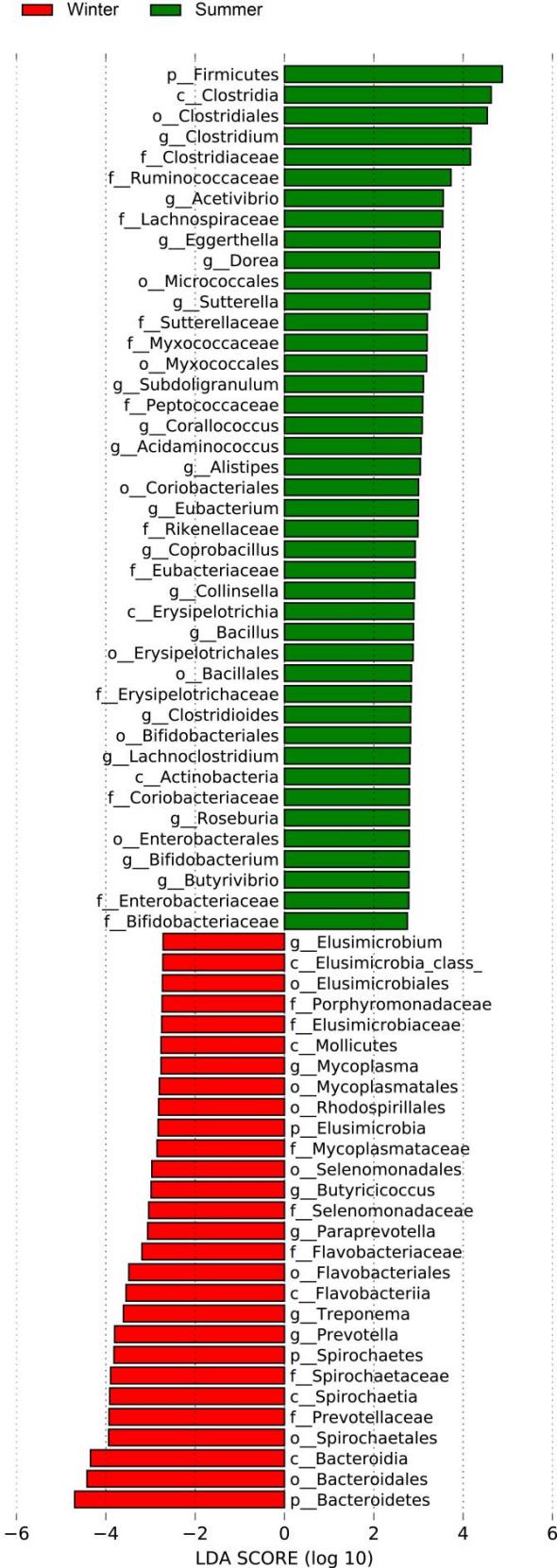


**Supplementary Figure S1.** Seasonal variations in gut microbiota composition. This figure illustrates the differential abundance of gut microbial taxa during summer (green bars) and winter (red bars). The LDA (Linear Discriminant Analysis) scores, presented on a logarithmic scale, reflect the relative abundance and statistical significance of each microbial taxon. Positive values indicate taxa that are more abundant in the summer, whereas negative values indicate taxa that are more abundant in the winter. Bars represent various microbial taxa categorized at different taxonomic levels: phylum (p), class (c), order (o), family (f), and genus (g).


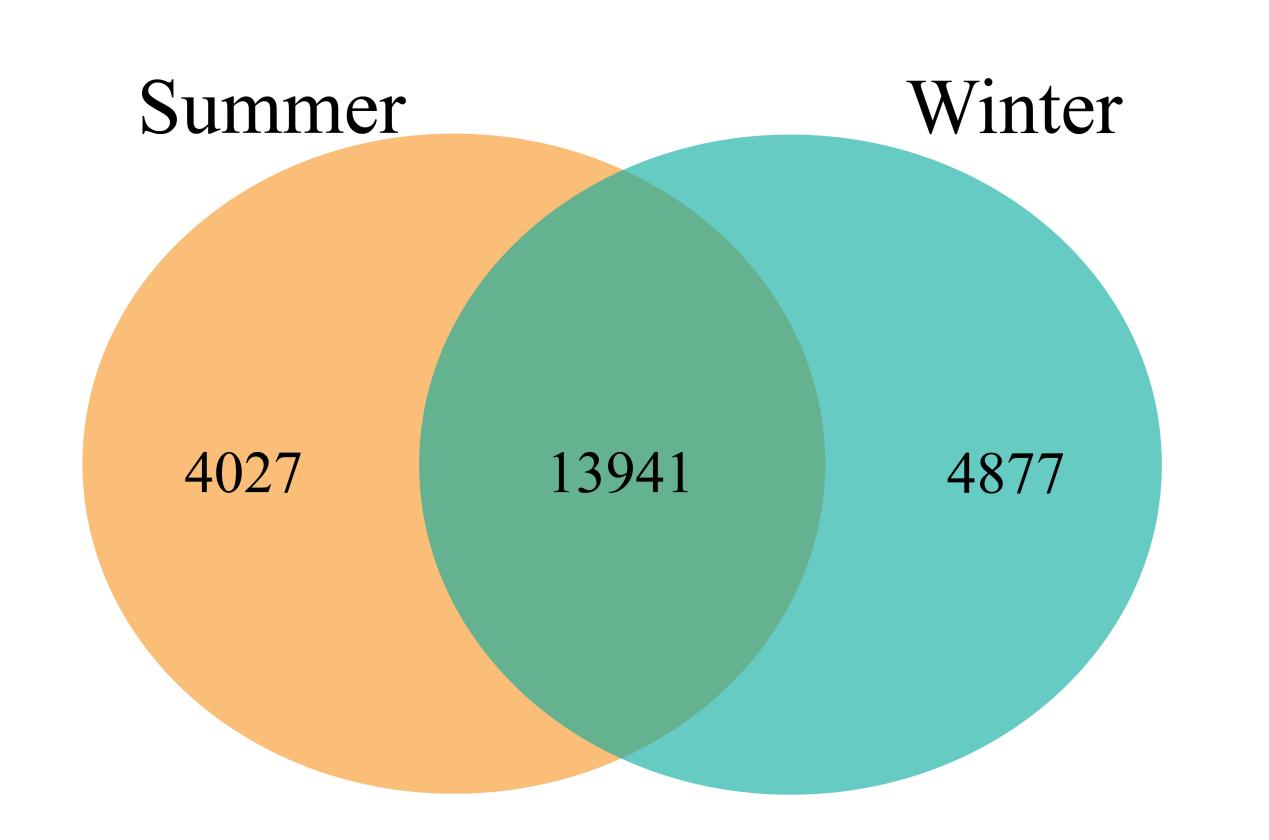


**Supplementary Figure S2.** The Venn diagram exhibits the distribution of unique and shared functional genes within the gut microbiota of Min pigs across the summer and winter seasons. A total of 4,027 genes were exclusive to the summer season (orange circle), while 4,877 genes were unique to winter (blue circle). The overlap represents 13,941 genes present in both seasons.


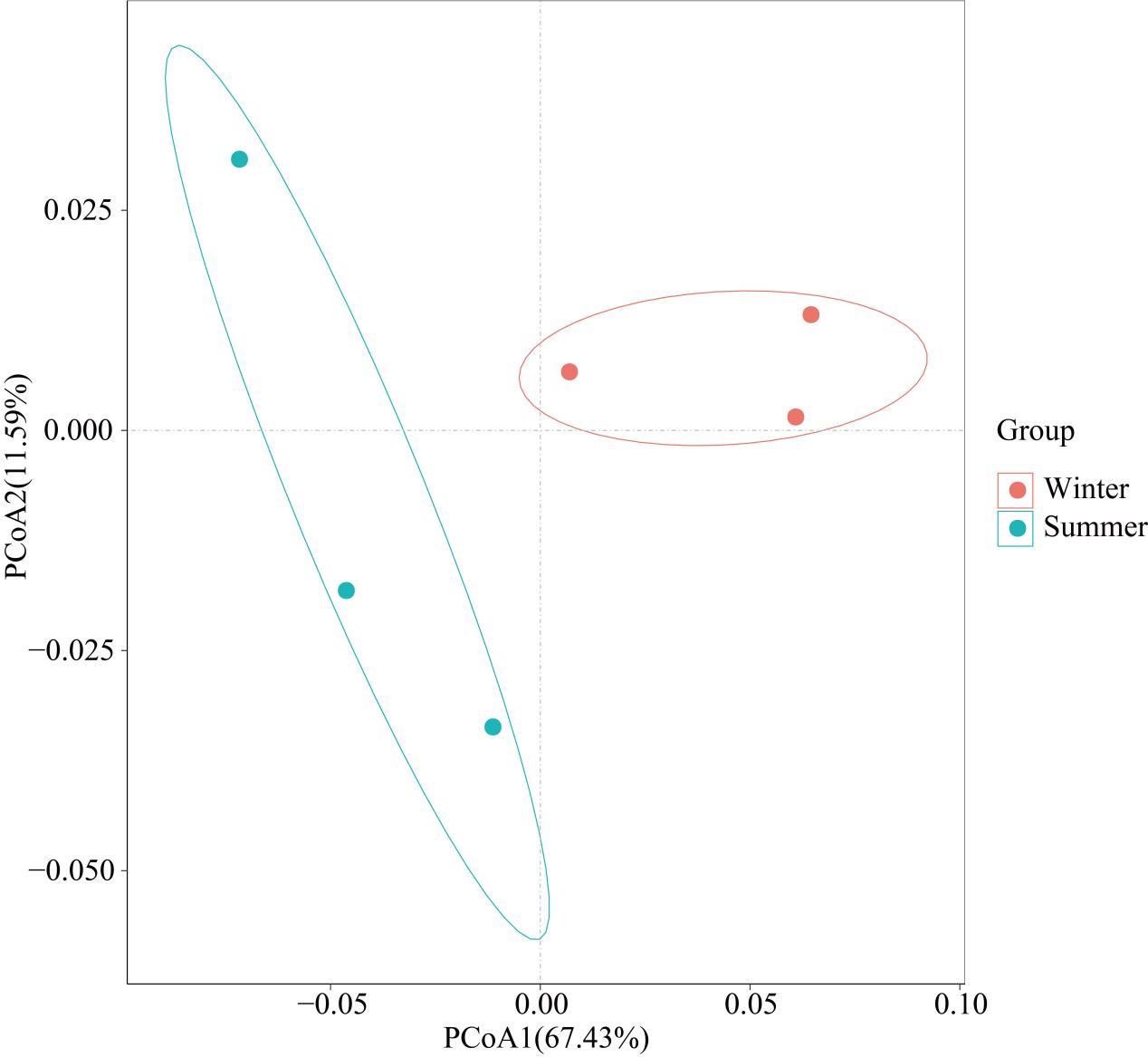


**Supplementary Figure S3.** Seasonal metabolic profiling in gut microbiota of Min Pigs via Principal Coordinates Analysis.


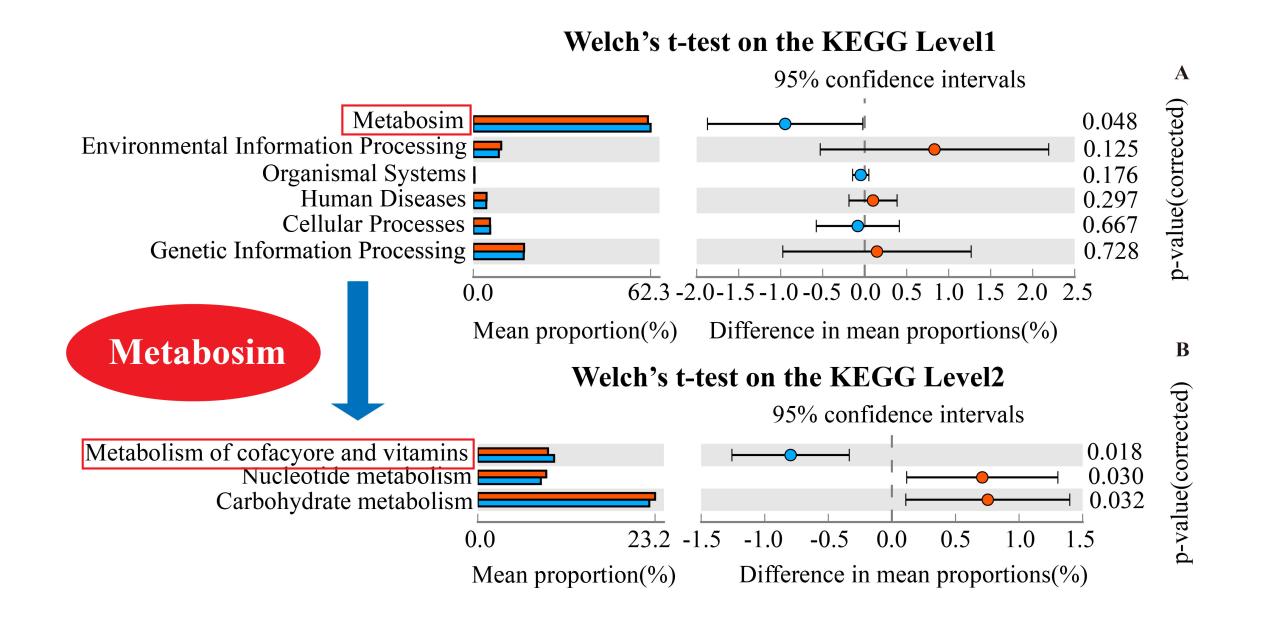


**Supplementary Figure S4.**  Seasonal variations of KEGG Pathways in gut microbiota of Min Pigs. (A) KEGG Level 1 pathways, with the “Metabolism” pathway showing a significant increase in winter compared to summer. (B) KEGG Level 2 within the “Metabolism” category, highlighting a substantial winter enrichment in the “Metabolism of cofactors and vitamins” pathway. Bars represent the mean proportion of each pathway with 95% confidence intervals.


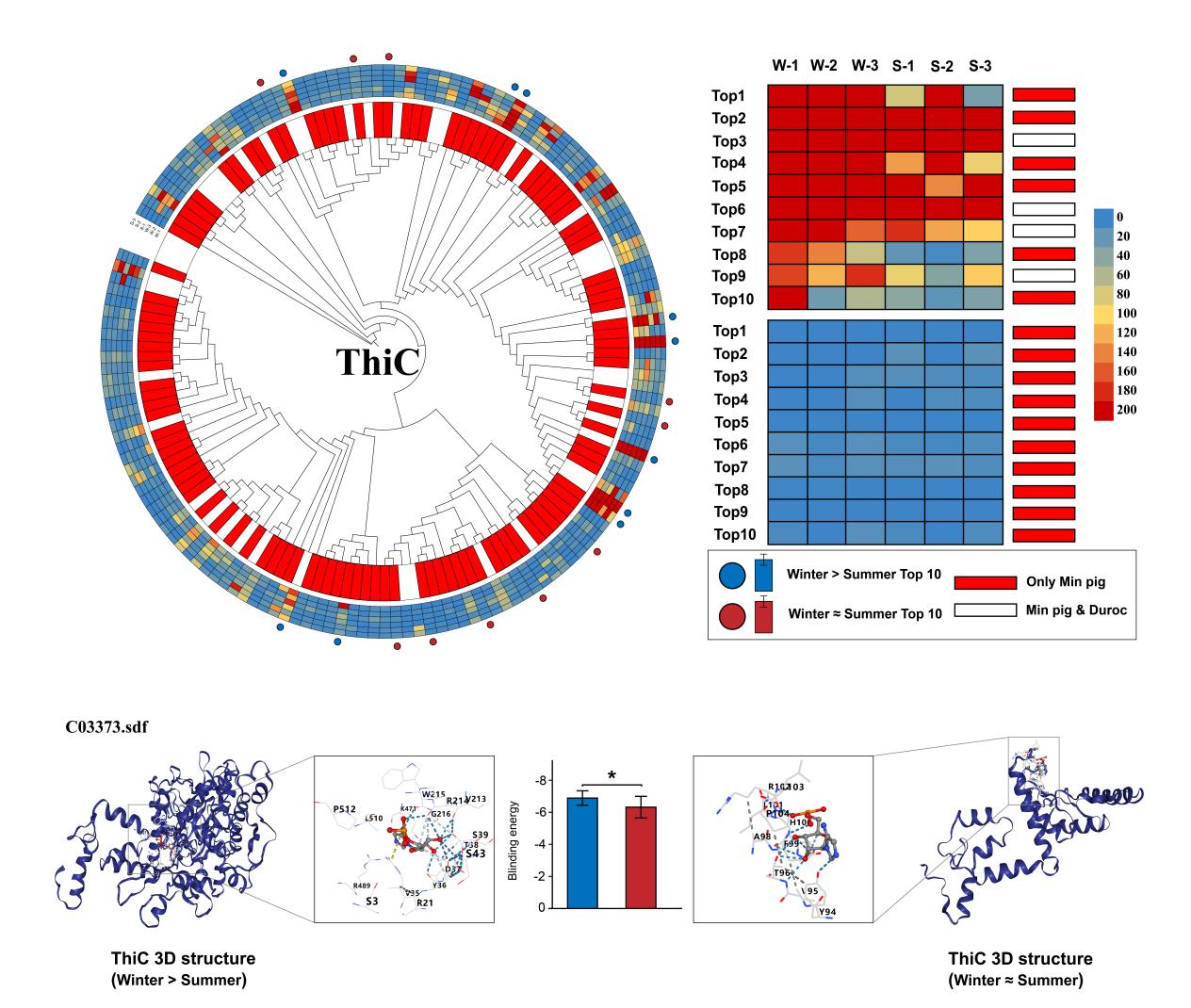


**Supplementary Figure S5.** Seasonal variation in *ThiC* gene sequences and protein binding efficiency. (A) Phylogenetic tree of *ThiC* sequences with orthologous gene pairs between Min pigs and Duroc pigs indicated in the inner circle. Unique sequences to Min pigs are marked in red, shared sequences in white. Sequence abundance across samples is represented in the outer circle, with the top ten sequences with the largest (red dot) and smallest (blue dot) winter-summer abundance disparities highlighted in the outermost bands. (B) The top ten sequences with the highest and lowest seasonal abundance changes, showing their abundance in winter (W-1, W-2, W-3) and summer (S-1, S-2, S-3) samples, along with their orthologous pairing in Min pigs and Duroc pigs. (C) Structural models and molecular docking simulations of the top ten sequences with the most significant and least significant seasonal abundance variation. On the left side, proteins with the highest binding affinities and most significant seasonal changes are shown. On the right side, proteins with the lowest affinities and least seasonal variation are displayed, emphasizing how seasonal shifts might affect protein function. Asterisks mark statistically significant differences (** *P* < 0.01, *** *P* < 0.001).


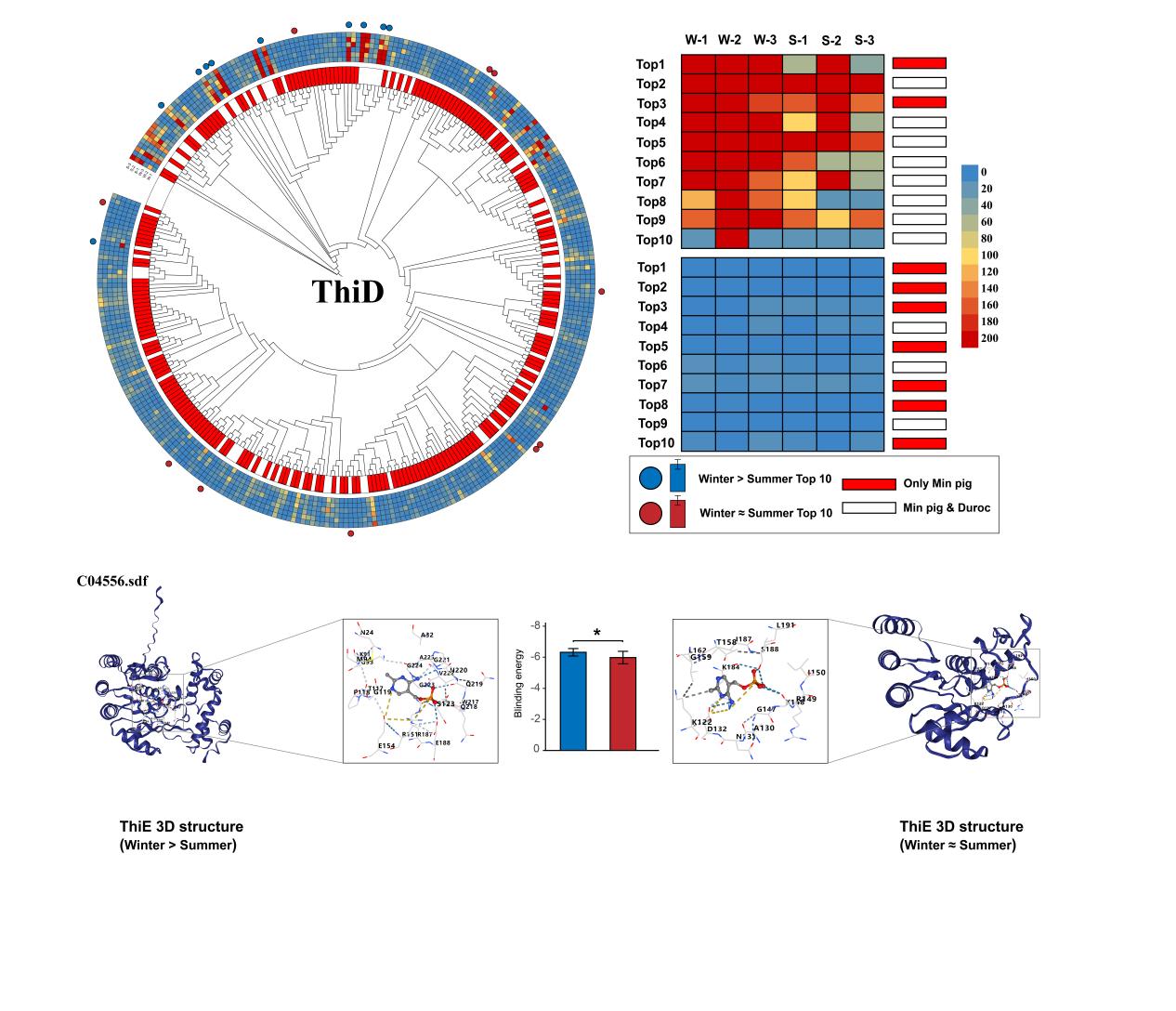


**Supplementary Figure S6.** Seasonal variation in *ThiD* gene sequences and protein binding efficiency. (A) Phylogenetic tree of *ThiD* sequences with orthologous gene pairs between Min pigs and Duroc pigs indicated in the inner circle. Unique sequences to Min pigs are marked in red, shared sequences in white. Sequence abundance across samples is represented in the outer circle, with the top ten sequences with the largest (red dot) and smallest (blue dot) winter-summer abundance disparities highlighted in the outermost bands. (B) The top ten sequences with the highest and lowest seasonal abundance changes, showing their abundance in winter (W-1, W-2, W-3) and summer (S-1, S-2, S-3) samples, along with their orthologous pairing in Min pigs and Duroc pigs. (C) Structural models and molecular docking simulations of the top ten sequences with the most significant and least significant seasonal abundance variation. On the left side, proteins with the highest binding affinities and most significant seasonal changes are shown. On the right side, proteins with the lowest affinities and least seasonal variation are displayed, emphasizing how seasonal shifts might affect protein function. Asterisks mark statistically significant differences (** *P* < 0.01, *** *P* < 0.001).


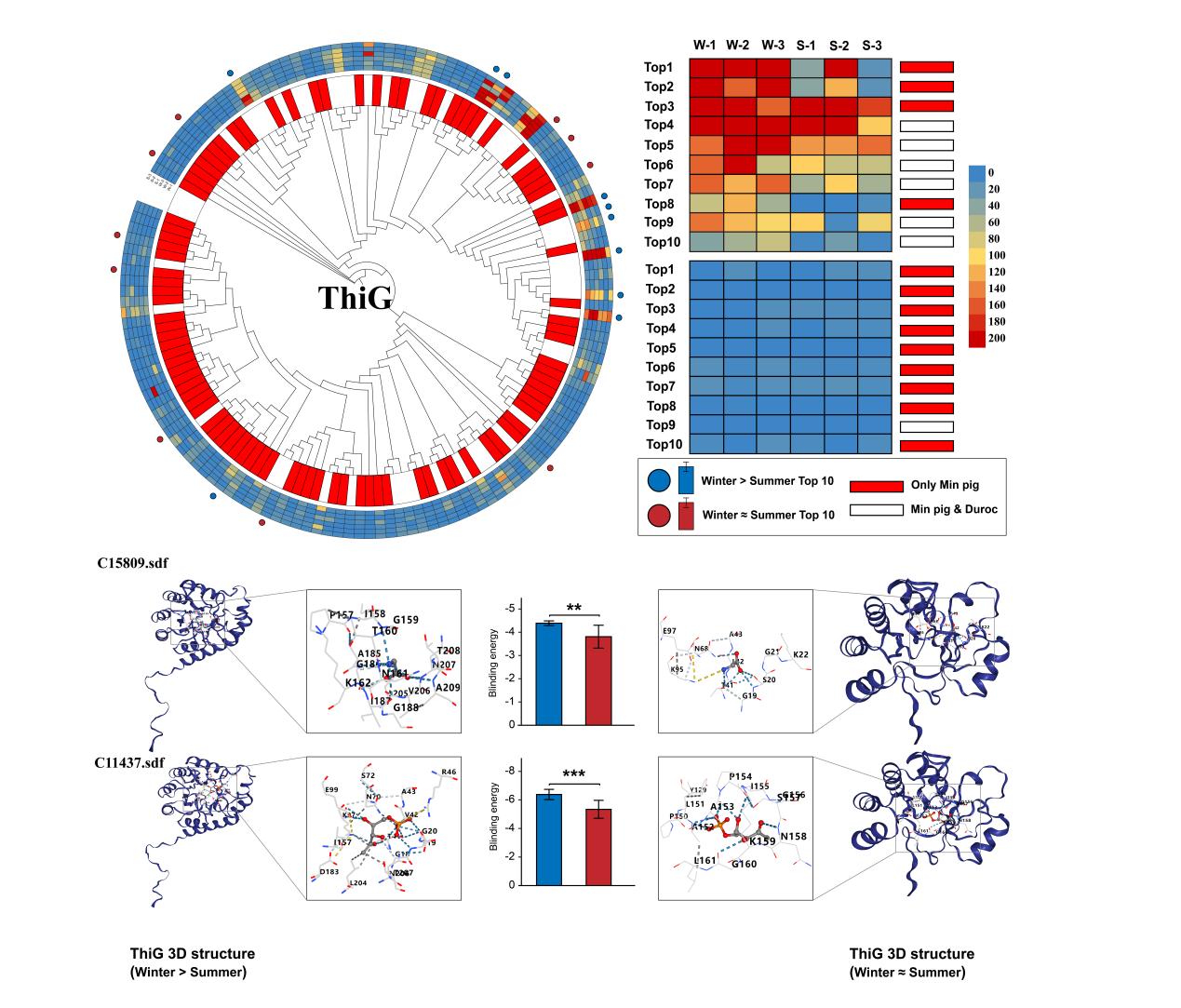


**Supplementary Figure S7.** Seasonal variation in *ThiG* gene sequences and protein binding efficiency. (A) Phylogenetic tree of *ThiG* sequences with orthologous gene pairs between Min pigs and Duroc pigs indicated in the inner circle. Unique sequences to Min pigs are marked in red, shared sequences in white. Sequence abundance across samples is represented in the outer circle, with the top ten sequences with the largest (red dot) and smallest (blue dot) winter-summer abundance disparities highlighted in the outermost bands. (B) The top ten sequences with the highest and lowest seasonal abundance changes, showing their abundance in winter (W-1, W-2, W-3) and summer (S-1, S-2, S-3) samples, along with their orthologous pairing in Min pigs and Duroc pigs. (C) Structural models and molecular docking simulations of the top 10 sequences with the most significant and least significant seasonal abundance variation. On the left side, proteins with the highest binding affinities and most significant seasonal changes are shown. On the right side, proteins with the lowest affinities and least seasonal variation are displayed, emphasizing how seasonal shifts might affect protein function. Asterisks mark statistically significant differences (** *P* < 0.01, *** *P* < 0.001).


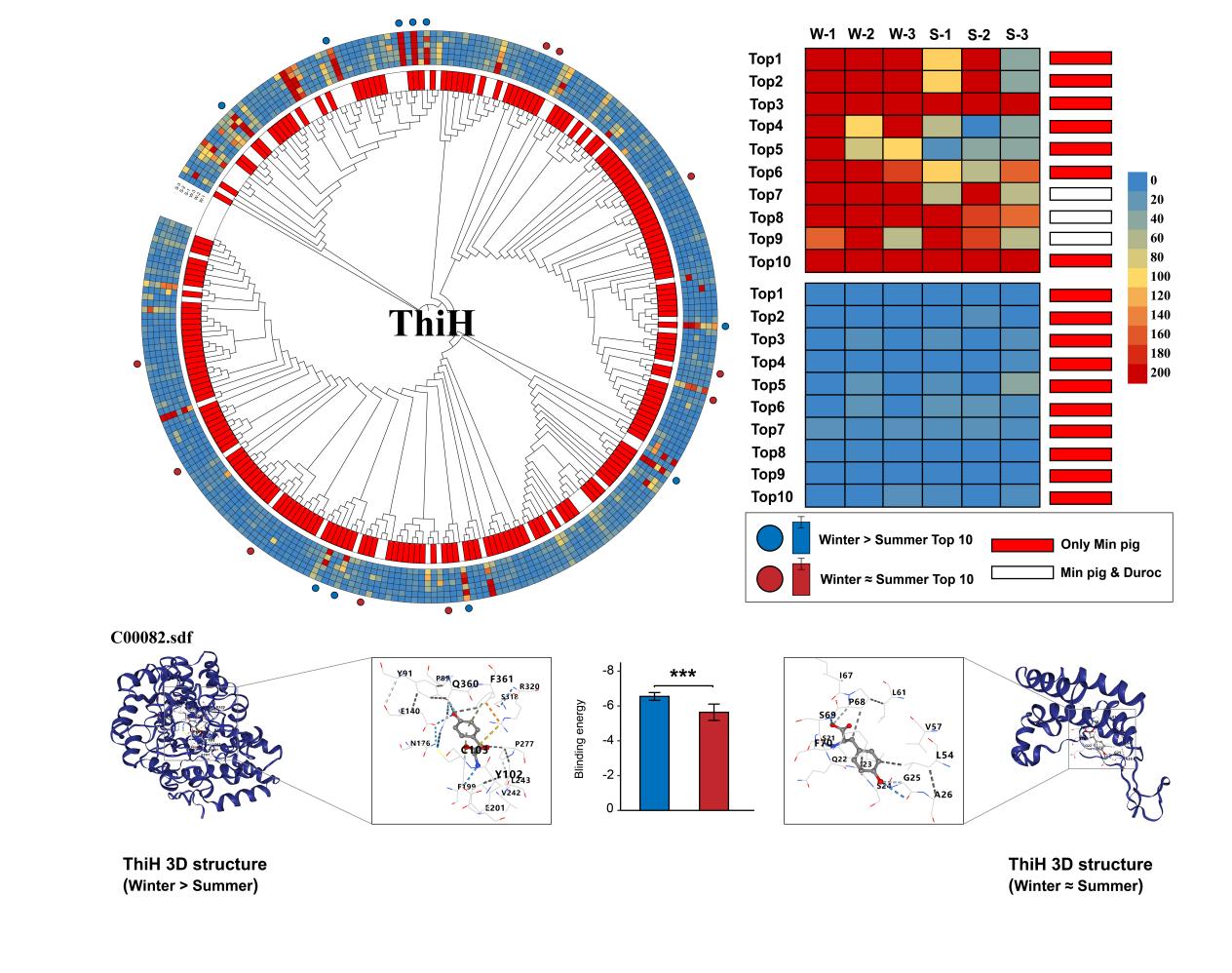


**Supplementary Figure S8.** Seasonal variation in *ThiH* gene sequences and protein binding efficiency. (A) Phylogenetic tree of *ThiH* sequences with orthologous gene pairs between Min pigs and Duroc pigs indicated in the inner circle. Unique sequences to Min pigs are marked in red, shared sequences in white. Sequence abundance across samples is represented in the outer circle, with the top ten sequences with the largest (red dot) and smallest (blue dot) winter-summer abundance disparities highlighted in the outermost bands. (B) The top ten sequences with the highest and lowest seasonal abundance changes, showing their abundance in winter (W-1, W-2, W-3) and summer (S-1, S-2, S-3) samples, along with their orthologous pairing in Min pigs and Duroc pigs. (C) Structural models and molecular docking simulations of the top ten sequences with the most significant and least significant seasonal abundance variation. On the left side, proteins with the highest binding affinities and most significant seasonal changes are shown. On the right side, proteins with the lowest affinities and least seasonal variation are displayed, emphasizing how seasonal shifts might affect protein function. Asterisks mark statistically significant differences (** *P* < 0.01, *** *P* < 0.001).


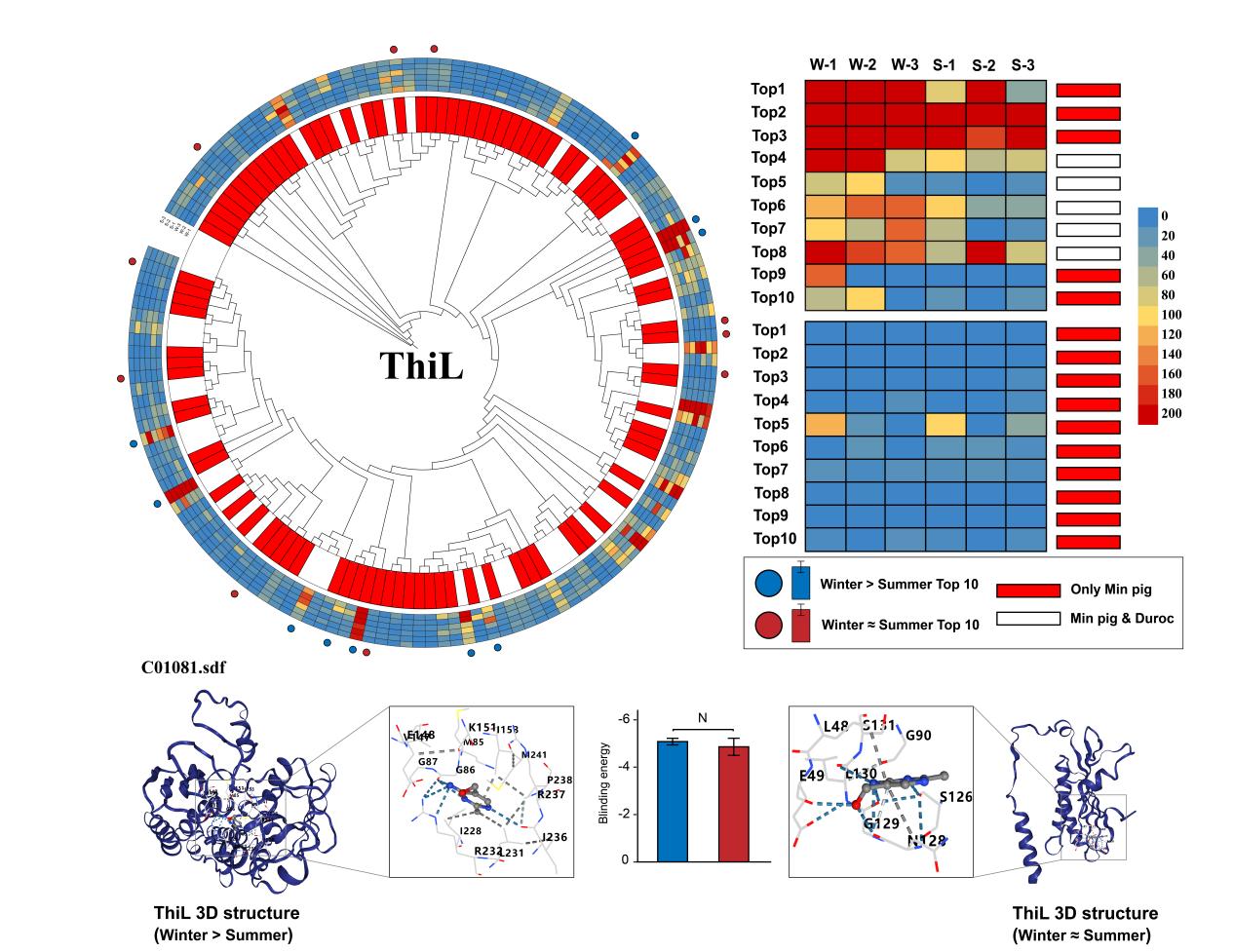


**Supplementary Figure S9.** Seasonal variation in *ThiL* gene sequences and protein binding efficiency. (A) Phylogenetic tree of *ThiL* sequences with orthologous gene pairs between Min pigs and Duroc pigs indicated in the inner circle. Unique sequences to Min pigs are marked in red, shared sequences in white. Sequence abundance across samples is represented in the outer circle, with the top ten sequences with the largest (red dot) and smallest (blue dot) winter-summer abundance disparities highlighted in the outermost bands. (B) The top ten sequences with the highest and lowest seasonal abundance changes, showing their abundance in winter (W-1, W-2, W-3) and summer (S-1, S-2, S-3) samples, along with their orthologous pairing in Min pigs and Duroc pigs. (C) Structural models and molecular docking simulations of the top ten sequences with the most significant and least significant seasonal abundance variation. On the left side, proteins with the highest binding affinities and most significant seasonal changes are shown. On the right side, proteins with the lowest affinities and least seasonal variation are displayed, emphasizing how seasonal shifts might affect protein function. Asterisks mark statistically significant differences (** *P* < 0.01, *** *P* < 0.001).
